# Supplementary material for: Netcombin: An algorithm for constructing optimal phylogenetic network from rooted triplets
Source: PLoS One. 2020 Sep 18;15(9):e0227842. doi: 10.1371/journal.pone.0227842 (PMC7500971; doi:10.1371/journal.pone.0227842)
Supplement: S4 File — (ZIP) [file pone.0227842.s004.zip › SequenceData-Triplet/sequenceData-10-20-30-0-Triplet/length 300, numseq 40, rate 10^-7/8 seq.pdf]

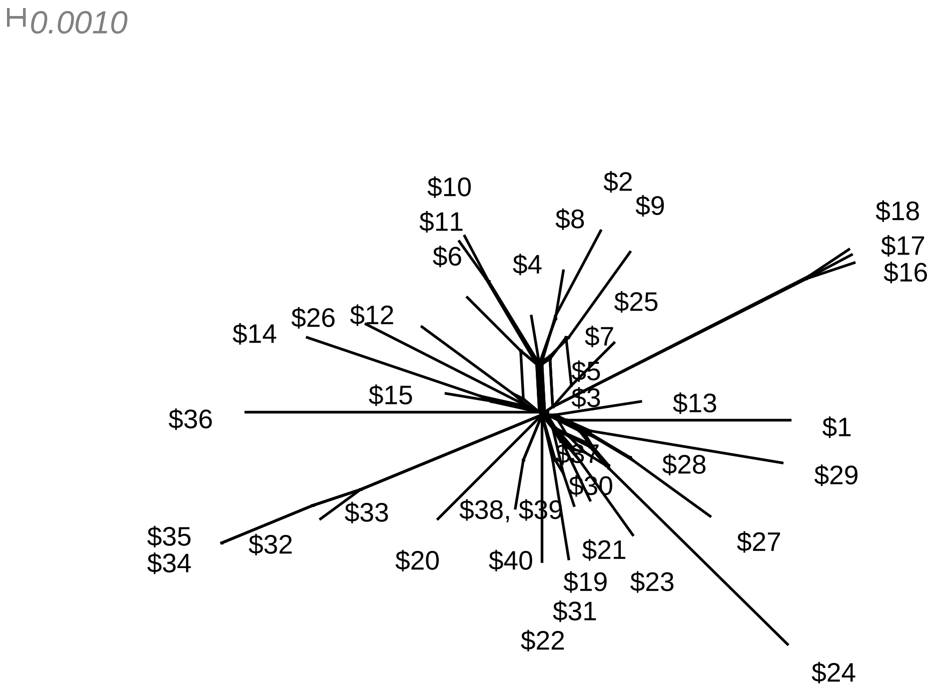
0.0010
$10
$2
$9
$18
$11
$8
$17
$6
$4
$16
$25
$26
$12
$14
$7
$5
$15
$3
$13
$36
$1
$37
$28
$29
$30
$33
$38, $39
$35
$32
$27
$34
$20
$40
$21
$19
$23
$31
$22
$24
